# 16S Metagenomics Reveals Dysbiosis of Nasal Core Microbiota in Children With Chronic Nasal Inflammation: Role of Adenoid Hypertrophy and Allergic Rhinitis

**DOI:** 10.3389/fcimb.2020.00458

**Published:** 2020-09-02

**Authors:** Massimiliano Marazzato, Anna Maria Zicari, Marta Aleandri, Antonietta Lucia Conte, Catia Longhi, Luca Vitanza, Vanessa Bolognino, Carlo Zagaglia, Giovanna De Castro, Giulia Brindisi, Laura Schiavi, Valentina De Vittori, Sofia Reddel, Andrea Quagliariello, Federica Del Chierico, Lorenza Putignani, Marzia Duse, Anna Teresa Palamara, Maria Pia Conte

**Affiliations:** ^1^Department of Public Health and Infectious Diseases, Microbiology Section, “Sapienza” University of Rome, Rome, Italy; ^2^Department of Pediatrics, Faculty of Medicine and Odontology, “Sapienza” University of Rome, Rome, Italy; ^3^Unit of Human Microbiome, Area of Genetics and Rare Diseases, Bambino Gesù Children's Hospital, IRCCS, Rome, Italy; ^4^Unit of Parasitology and Area of Genetics and Rare Diseases, Unit of Human Microbiome, Department of Laboratories, Bambino Gesù Children's Hospital, IRCCS, Rome, Italy; ^5^Department of Public Health and Infectious Diseases, “Sapienza” University of Rome, Laboratory Affiliated to Istituto Pasteur Italia – Fondazione Cenci Bolognetti, San Raffaele Pisana, IRCCS, Rome, Italy

**Keywords:** nasal microbiota, allergic rhinitis, adenoid hypertrophy, core microbiota, chronic inflammation

## Abstract

Allergic rhinitis (AR) and adenoid hypertrophy (AH) are, in children, the main cause of partial or complete upper airway obstruction and reduction in airflow. However, limited data exist about the impact of the increased resistance to airflow, on the nasal microbial composition of children with AR end AH. Allergic rhinitis (AR) as well as adenoid hypertrophy (AH), represent extremely common pathologies in this population. Their known inflammatory obstruction is amplified when both pathologies coexist. In our study, the microbiota of anterior nares of 75 pediatric subjects with AR, AH or both conditions, was explored by 16S rRNA-based metagenomic approach. Our data show for the first time, that in children, the inflammatory state is associated to similar changes in the microbiota composition of AR and AH subjects respect to the healthy condition. Together with such alterations, we observed a reduced variability in the between-subject biodiversity on the other hand, these same alterations resulted amplified by the nasal obstruction that could constitute a secondary risk factor for dysbiosis. Significant differences in the relative abundance of specific microbial groups were found between diseased phenotypes and the controls. Most of these taxa belonged to a stable and quantitatively dominating component of the nasal microbiota and showed marked potentials in discriminating the controls from diseased subjects. A pauperization of the nasal microbial network was observed in diseased status in respect to the number of involved taxa and connectivity. Finally, while stable co-occurrence relationships were observed within both control- and diseases-associated microbial groups, only negative correlations were present between them, suggesting that microbial subgroups potentially act as maintainer of the eubiosis state in the nasal ecosystem. In the nasal ecosystem, inflammation-associated shifts seem to impact the more intimate component of the microbiota rather than representing the mere loss of microbial diversity. The discriminatory potential showed by differentially abundant taxa provide a starting point for future research with the potential to improve patient outcomes. Overall, our results underline the association of AH and AR with the impairment of the microbial interplay leading to unbalanced ecosystems.

## Introduction

The human nasal cavity hosts a complex bacterial community that is mainly stable at the genus level but can vary between individuals and in relation to the seasonal allergens (Valero et al., 2017). However, the spatial heterogeneity of the nasal epithelium is not homogenous, but instead has different levels of keratinization. The anterior nares, in particular, covered by desquamated fully keratinized dead cells, tend to be cooler than other nasal sites and the sebaceous glands percolate through these regions (Yan et al., 2013). These micro-environmental differences may give rise to spatial patterns in community structure (Krismer et al., 2017; Proctor and Relman, 2017). Culture-independent analysis of 16S rRNA gene sequences revealed that the nasal microbiota of healthy subjects consists primarily of members of the phyla *Actinobacteria, Firmicutes*, and *Proteobacteria* (Costello et al., 2009; Grice et al., 2009; Camarinha-Silva et al., 2012; Wos-Oxley et al., 2016). However, bacterial communities in the nares of children differed strikingly from adults. *Proteobacteria, Firmicutes, Bacteroidetes, Actinobacteria*, and *Fusobacteria* represent the five most predominant phyla. On a lower taxonomic level, the most prevalent genera were *Moraxella, Haemophilus, Streptococcus*, and *Flavobacterium*. Other fairly common genera were *Dolosigranulum, Corynebacterium, Neisseria*, and *Fusobacterium* (Bogaert et al., 2011; Oh et al., 2012; Camarinha-Silva et al., 2014). Allergic and inflammatory diseases of the airways such as allergic rhinitis (AR), chronic rhinosinusitis (CRS), and asthma are common chronic diseases that affect the quality of life of patients. The incidence of these inflammatory diseases appears to be increasing in most countries, and people living in urban and industrialized areas suffer more frequently from these conditions than those living in rural areas (D'Amato, 2002; González-Díaz et al., 2016). Several researches have shown evidence for dysbiosis of the nasal microbiota in the context of allergic and infective inflammation of the airways (Abreu et al., 2012; Hoggard et al., 2017; Lal et al., 2017; Fazlollahi et al., 2018). However, there are no studies regarding AR and adenoid hypertrophy (AH), that are common pathologies in children and are often associated with each other. AR is a nasal allergic disease characterized by systemic and nasal overproduction of immunoglobulin E (IgE) skewing of mucosal immune homeostasis toward a TH2-type response, and recall of eosinophils and mast cells (Hyun et al., 2018). AH is characterized by hypertrophy of the adenoid tonsil, as a consequence of different antigenic stimulation triggering chronic inflammation (Sakarya et al., 2017). AR and AH cause the obstruction and the increase in airflow resistance in the upper airway (Ballikaya et al., 2018). AH condition, in particular, is involved in the development of obstructive sleep apnea (OSA) (Huang and Guilleminault, 2017), leading to poor quality of life. When both coexist, the clinical symptoms are similar but obstructive complications and snoring could prevail (Cao and Xu, 2019). Moreover, the prevalence of allergic diseases in children seems to be increasing in recent times (Zou et al., 2018), and the most significant causes of the increase are thought to be changes in environmental factors, especially air pollution (Mady et al., 2018). Recently it has been showed that exposure to air pollutants influences microbiota and alters the normal homeostasis within the bacterial community (Mariani et al., 2018). To date, in spite of the growing level of interest by the scientific community, very little is known on the relationship between the nasal mucosal microenvironment, nasal allergic inflammation and the nasal microbiota. This study aimed to identify the community structure, functional potential and bacterial interplay of the nasal microbiota in pediatric healthy subjects as well as in children with AR and AH. The primary outcome of this study was to evaluate the composition of the commensal microbiota in the anterior nares of pediatric subjects suffering from AR, AH or both diseases. The secondary outcome was to study the correlation between the nasal flow and the microbial composition in the anterior nares of these children.

## Materials and Methods

### Subjects and Study Design

We consecutively enrolled children between 6 and 12 years old in the outpatient setting of pediatric allergology of Umberto I Hospital in Rome from December 2016 to November 2017. Children with nasal obstruction caused by allergic sensitization or adenotonsillar hypertrophy or both were enrolled in the AR, AH, or AR + AH groups, respectively. At the enrollment visit, children with AR and AR + AH presented only the allergic sensitizations showed in Table 1. Children without nasal obstruction who were in the allergy control service were enrolled as controls. Exclusion criteria were the presence of chronic pathologies (asthma, genetic diseases, cardiovascular and/or pulmonary diseases, craniofacial malformations) or acute, as well as the use of drugs (steroids or systemic or local antihistamines, antibiotics), in progress and/or in the month preceding the study. The diagnosis of AR was formulated according to standardized criteria of Allergic Rhinitis and its Impact on Asthma (ARIA) (Brozek et al., 2017) and that of adenoid hypertrophy with the evaluation of fiber-like grading according to Cassano et al. (2003). The study protocol was approved by Bioethics Committee of the “Sapienza” University of Rome, (registration number 3419, registered 12 November 2014). At the visit, children performed a complete routine clinical examination, nasal fiberoptic endoscopy (NFE), active anterior rhinomanometry (AAR), and microbiological evaluation of nasal cavities with nasal swabs.

**Table 1:** Characteristics of the studied population.

	**CTRL (No. = 13)**	**AH (No. = 20)**	**AR (No. = 29)**	**AH+AR (No. = 13)**	***p*-value**
Sex (M/F) (No.)	7/6	10/10	20/9	8/5	0.568
Age, (years), (mean ± SD)	8.0 ± 2.2	8.0 ± 1.5	9.1 ± 2.0	7.9 ± 1.3	0.072
Season (FW/SS) (No.)	8–5	9–11	18–11	7–6	0.659
mNF% (mean ± SD)	94.3 ± 7.0	55.6 ± 13.0	68.0 ± 21.4	51.4 ± 12.0	<0.0001^a^^,^ ^b^^,^ ^c^^,^ ^f^
Antigen-specific serum IgE (UA/mL)					
*D. pteronyssinus* (mean ± SD)	0.0 ± 0.0	0.0 ± 0.0	29.6 ± 38.3	6.7 ± 11.4	<0.0001^b^^,^ ^c^^,^ ^d^^,^ ^e^
*D. farinae* (mean ± SD)	0.0 ± 0.0	0.0 ± 0.0	30.3 ± 38.1	7.0 ± 14.3	<0.0001^b^^,^ ^c^^,^ ^d^^,^ ^e^
*C. dactylon* (mean ± SD)	0.0 ± 0.0	0.0 ± 0.0	7.8 ± 20.5	12.1 ± 20.1	<0.0001^b^^,^ ^c^^,^ ^d^^,^ ^e^
*L. perenne* (mean ± SD)	0.0 ± 0.0	0.0 ± 0.0	16.7 ± 32.8	20.5 ± 38.8	<0.0001^b^^,^ ^c^^,^ ^d^^,^ ^e^
*Parietaria* (mean ± SD)	0.0 ± 0.0	0.0 ± 0.0	7.2 ± 20.8	0.1 ± 0.3	0.016^b^^,^ ^d^
*Alternaria* (mean ± SD)	0.0 ± 0.0	0.0 ± 0.0	1.3 ± 4.2	0.1 ± 0.4	0.017^b^^,^ ^d^
*O. europea* (mean ± SD)	0.0 ± 0.0	0.0 ± 0.0	4.7 ± 9.9	0.7 ± 1.1	0.000^b^^,^ ^c^^,^ ^d^^,^ ^e^

M, male; F, female; FW, fall/winter; SS, spring/summer; D. pteronyssinus, Dermatogagoide pteronyssinus; D. farinae, Dermatofagoide farinae; C. dactylon, Cynodon dactylon; L. perenne, Lolium perenne; O. europea, Olea europea;

a p ≤ 0.05 AH vs. Controls;

b p ≤ 0.05 AR vs. CTRL;

c p ≤ 0.05 AH + AR vs. CTRL;

d p ≤ 0.05 AH vs. AR;

e p ≤ 0.05 AH vs. AH + AR;

f *p ≤ 0.05 AR vs. AH + AR*.

### Antigen-Specific IgE

Antigen-specific IgE levels were analyzed in the blood of enrolled children using immunological assays with ImmunoCAP Fluorescence Enzyme Immuno Assay (Phadia AB) in a clinical laboratory under contract. *Cynodon dactylon, Lolium perenne, Dermatophagoides farinae, Dermatophagoides pteronyssinus, Parietaria, Alternaria*, and *Olea europea* were detected as specific allergens. The results of the ImmunoCAP system were given as exact values in UA/mL. Values of 0.35 UA/mL or higher were taken as a sign of sensitization (Yamamoto-Hanada et al., 2017).

### Nasal Fiberoptic Endoscopy

NFE was performed by an independent expert pediatric otorhinolaryngologist using a 2, 7 mm diameter endoscope. The degree of AH was assessed according to Cassano's criteria, considering in the group AH children with adenoids occluding the upper half of the choanal opening for more than 25%.

### Active Anterior Rhinomanometry

Children wore a face mask, closed and breathed only through the nose in accordance with the International Committee on Standardization of Rhinomanometry (Clement, 1984). In accordance with Zapletal and Chalupová (2002), the degree of nasal obstruction, based on rhinomanometry test values, was estimated as fraction of predicted values (p.v.) of mean nasal flow (mNF). The presence of nasal obstruction corresponded to a mean nasal flow inferior to 77%.

### Nares Sampling and DNA Preparation

Samples were obtained from the anterior nares of external patients and healthy individuals (CTRL) in the Pediatric Department at Hospital “Policlinico Umberto I” “Sapienza” University of Rome. The nasal swabs were inserted into each nostril, rotated for five times applying constant pressure and placed immediately into a sterile tube. Samples were immediately transported to the microbiology laboratory where they were kept at −80°C until further processing. In order to maximize bacterial recovery, swab samples were gradually led to 4°C and kept overnight in a solution of Phoshate Buffered Saline 0.01 M (Sigma-Aldrich). At the end of the incubation period, samples were vigorously vortexed for about 5 min and subsequently centrifuged at 12.000 rcf for 5 min. DNA extraction was performed on pelleted bacteria by using QIAamp DNA Mini Kit (Qiagen, Germany) according to the manufacturer's instruction.

### 16S Ribosomal RNA Gene Next Generation Sequencing

Amplification of the variable region V3–V4 from the 16S rRNA gene (460 bp) was carried out using the primer pairs 16S_F 50 -(TCG TCG GCA GCG TCA GAT GTG TAT AAG AGA CAG CCT ACG GGN GGC WGC AG)-30 and 16S_R 50 -(GTC TCG TGG GCT CGG AGATGT GTATAA GAG ACA GGA CTA CHV GGG TAT CTA ATC C)-30 described in the MiSeq rRNA Amplicon Sequencing protocol (Illumina, San Diego, California, USA). The PCR reaction was set up following the protocol from the two KAPA Hifi HotStart ready Mix kit (KAPA Biosystems Inc., Wilmington, Massachusetts, USA). DNA amplicons were cleaned-up by AMPure XP beads (Beckman Coulter Inc., Beverly, Massachusetts, USA). A second amplification step was performed to obtain a unique combination of bar-coded Illumina Nextera forward and reverse adaptor-primers. The final library was cleaned-up using 50 ml of AMPure XP beads, quantified using QuantiT PicoGreen dsDNA Assay Kit (Thermo Fisher Scientific, Waltham, Massachusetts, USA) and diluted in equimolar concentrations (4 nmol/l). To generate paired-end reads of 300 base-length, samples were pooled together before sequencing on the Illumina MiSeqTM platform according to manufacturer's specifications.

### Quality Control of the Sequences and OTU Picking

After demultiplexing, reads were merged by using USEARCH v11 (Edgar, 2010) with a minimum percentage identity of 90% between aligned sequences. Subsequently, the primer sequences were removed by using Cutadapt 2.1 (Martin, 2011) and the sequences were filtered in order to keep only those presenting a total expected error ≤0.8 and a size over a range of 400–460 bp. Sequences that passed the quality filter were imported in the software package Quantitative Insights into Microbial Ecology 2 (QIIME2) V18.6 (Bolyen et al., 2019) and passed to the Dada2 algorithm (Callahan et al., 2016) for chimera-checking. QIIME2 was used for all downstream analyses except those for which an alternative software package is clearly indicated. Operational taxonomic units (OTUs) defined by a 97% of similarity were picked by clustering sequences with an open reference approach against the 97% clustered Greengenes rDNA reference database v13_8. In order to minimize artifact, OTUs found in only one sample and/or presenting <10 sequences across the whole population were filtered out.

### Alpha and Beta-Diversity Analysis

**Figure 1:**
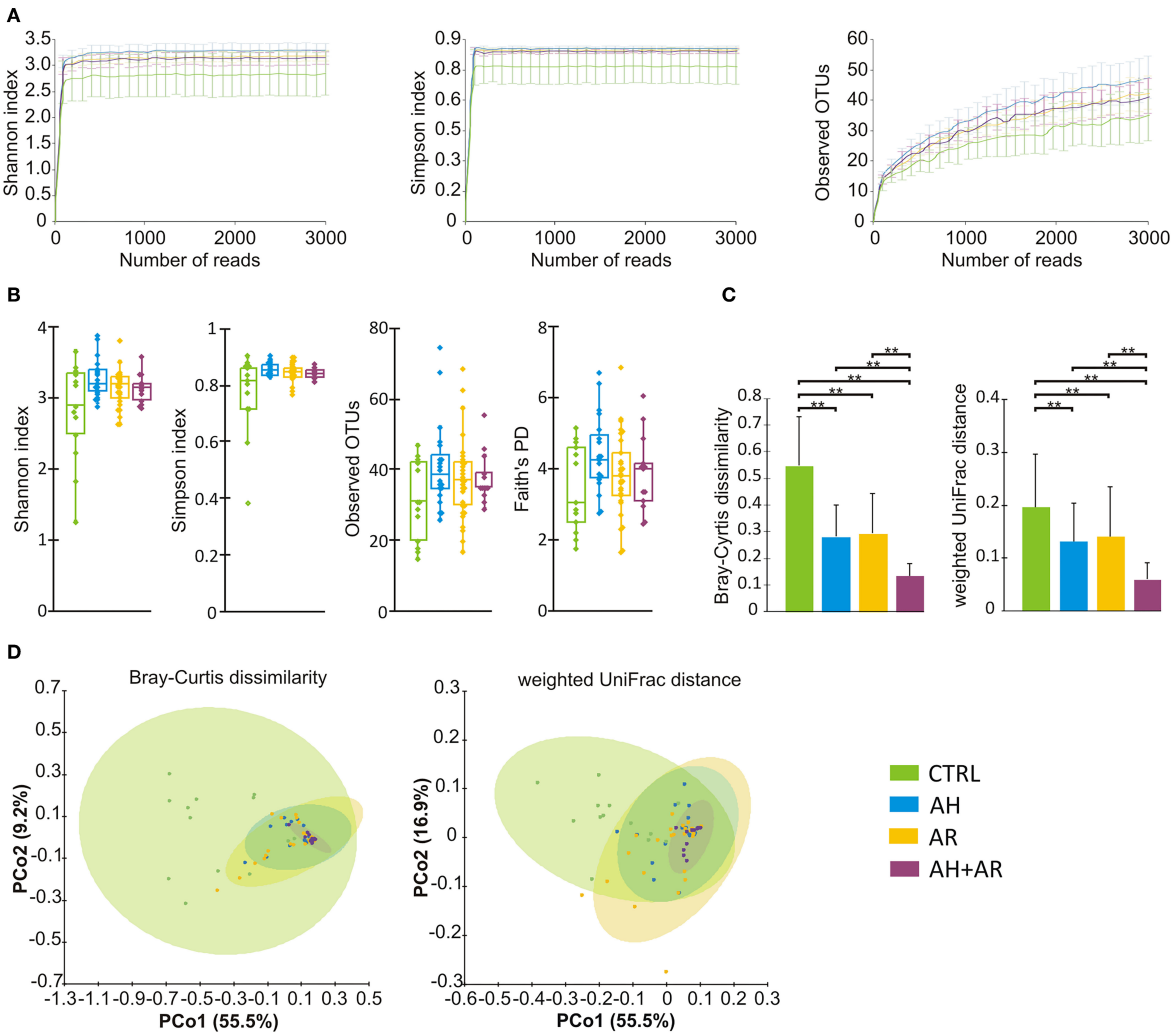
Microbiota diversity analysis. **(A)** Color coded rarefaction curves. For each group, the average values of α-diversity indexes with 95% confidence intervals were reported at different sequencing depth **(B)** box plots showing α-diversity estimators, measured for each groups. **(C)** Barplots relative to the intra-group distribution of the considered β-diversity estimators among groups. Data are expressed as mean ± SD while the presence of statistically significant differences was determined by Kruskal-Wallis rank sum test followed by pairwise Dunn's *post-hoc* tests. **(D)** PCoA plot of bacterial β-diversity based on Bray-Curtis dissimilarity and weighted UniFrac distance according to individual health status. For each group, the 95% confidence interval has been drawn. Numbers between parenthesis represents the percentage of the total variance explained by the principal coordinates. Where needed, the computed *p*-values were corrected by using the FDR procedure to take into account for multiple comparisons. A *p*-value ≤ 0.05 was considered statistically significant. **p < 0.0001.

Samples were rarefied to a total of 2,000 reads in agreement to the rarefaction curves computed for each considered α diversity index (Figure 1A). To take into account different components of diversity such as richness, evenness, and phylogenetic distance, four diversity indices (Shannon, Simpson, number of observed OTUs, Faith's phylogenetic distance), were calculated as metrics for α-diversity, while two different distance metrics (Bray-Curtis, weighted-UniFrac) were used for β-diversity. PCoA was performed to compare the overall composition of the bacterial community within samples.

### Taxonomy Assignment

The taxonomy assignment of OTUs was carried out by using a Naive Bayes classifier trained on a custom 97% clustered version of the Greengenes rDNA v13_8 reference database in which the sequences have been trimmed to only include the V3-V4 regions. OTUs not identified at species level and/or identified with a confidence <0.75, using the reference database, were assigned to the deepest taxonomic level on the base of BLAST results obtained by querying sequences against available published data and taking only those results in agreement to the taxonomy assigned by the Naive Bayes classifier approach if a confidence >0.75 were determined.

### Network Analysis

For each phenotype, a correlation network was computed independently. Briefly, a first filtering step was performed by removing OTUs present with a mean relative abundance of <0.01% across all population. Subsequently, for each separate group, OTUs with a count of zero were filtered out and the remaining unique entries were tested for their correlations by using CoNet v1.1.1 (Faust and Raes, 2016) available as an application in Cytoscape (Shannon et al., 2003). The following combination of methods, Pearson correlation, Spearman rank sum correlation, Bray-Curtis dissimilarity, and Kullback-Leibler divergence, was used with a cutoff threshold of 0.4 for both positive and negative values (−0.4 ≥ correlation ≥ 0.4) for all considered metrics in order to overcome weakness presented by the use of a single metric respect to compositionality, matching zeros and sample size. Only correlations supported by at least two different correlation metrics were retained. The Statistical significance of each pair was tested using 500 row shuffle randomizations followed by 100 bootstraps. The *p-*values relative to multi-edges connecting the same node pair were merged using the Fisher's method and the merged *p*-values were corrected for multiple comparisons. In each randomization round a sample-wise normalization step was performed for each item pair in order to account for compositionality bias. Topological parameters were calculated for each computed network by using the Network Analyzer plugin included in Cytoscape.

### Putative Functional Profiling

Functional metagenomes were predicted from 16S rDNA reads using the Phylogenetic Investigation of Communities by Reconstruction of Unobserved State (PICRUSt) (Langille et al., 2013). Since the PICRUSt method accepts only OTU with GreenGene OTU identifier, all OTUs to which a compatible identifier was not assigned by the open reference OTU picking methods were filtered out. The resulting OTU table was used as the base for the prediction of metagenomes, which were functionally categorized based on KEGG pathways at L2 level of classification.

### Statistical Analysis

Chi square test followed by Fisher's exact *post hoc* test was used for assessment of the association of frequencies among groups while Mann-Whitney *U*-test, as well as Kruskal-Wallis test followed by Dunn's *post hoc* test, were performed to determine significant differences respect to continuous variables. The Games-Howell test implemented in the Statistical analysis of taxonomic and functional profiles (STAMP) software v2.1.3 (Parks et al., 2014) was used as *post hoc* test for the differential abundant analysis of taxa. The permutational multiple analysis of variance (PERMANOVA) test with 1,000 permutations was calculated, in QIIME2, on beta diversity distance matrices to assess the presence of statistically significant partitions between AR, AH, AH+AR, and CTRL groups. The Spearman's rank correlation coefficient was calculated in order to evaluate the presence of statistically significant cross-correlations between the relative abundance of taxa and clinical variables (mNF%; Antigen-specific IgE levels), across the whole population of studied subjects. Obtained results were reported as separate heatmaps for each considered taxonomic level. ROC curves were computed for differentially abundant taxa in order to assess their ability to discriminate subjects according to their health status. The AUC was calculated by using the Hanley and McNeil method while the presence of statistically significant differences respect to AUC 0.5 (random classifier) was performed by using the Student *t*-test. An AUC = 0.7 was considered as threshold to determine a good discrimination model. The linear discriminant analysis (LDA) effect size (LEfSe) method (Segata et al., 2011) was performed to identify differentially abundant microbial functional pathways among groups (α = 0.05 after FDR correction, LDA score > 3.0). Statistical calculations, for which a software was not explicitly indicated, were performed by using XLstat software v 2016.02.28451 (Statsoft, USA) or R statistical environment version v3.2.5 (https://cran.r-project.org). The Benjamini–Hochberg false discovery rate (FDR) correction was used to account for multiple hypothesis testing when necessary. In all cases, a *p*-value ≤ 0.05 was considered statistically significant.

## Results

A total of 75 pediatric subjects (mean age 8.4 ± 1.8 years) were enrolled of which 20, 29, and 13 were diagnosed for AH, AR, and both pathologies, respectively while 13 were controls. Concerning the distribution of sex, age, and season of sampling, no statistically significant differences were found among groups (Table 1). Mean nasal flow percentage (mNF%) was significantly lower in diseased groups compared to the controls and in AH+AR subjects respect to AR ones. On a total of 42 subjects positively diagnosed for AR, the 85.7% (36/42) were sensitive to almost two tested antigens while only 14.3% (6/42) showed sensitization to only one. As expected, the tested antigen-specific serum IgE showed significantly higher values in groups with allergic rhinitis compared to those controls and AH group, while no differences were found between the AR group and the AH+AR one. Regarding sensitization to *Parietaria* and *Alternaria*, obtained results did not show differences between the AH + AR group and the controls or the AH subjects even if we cannot exclude that the negative results should depend on the limited number of tested sampled. All 75 samples underwent 16s rRNA gene-based microbiota analysis and resulted to be eligible for following bioinformatic downstream analysis. From a total of 3.307.515 sequences passing the filtering bioinformatics processes (mean ± SD, 44100.2 ± 18869.6/samples), 429 OTUs were identified.

### AH and AR Patients Show Similar Alterations in Nasal Microbiota

With regard to the α-diversity, although no statistically significant differences were found among phenotypes, the control group tended to show lower biodiversity values as well as higher variability as determined by the wider interquartile range (IQR) (Figure 1B). Regarding β-diversity, the Principal Coordinates Analysis (PCoA) determined statistically significant separations for both Bray Curtis dissimilarity and weighted UniFrac distance across all different phenotypes (Bray Curtis, *p* = 0.001; weighted UniFrac, *p* = 0.001). The pairwise comparisons between groups evidenced differences in microbiota composition between CTRL and AR (Bray Curtis, *p* = 0.002; weighted UniFrac, *p* = 0.002), AH (Bray Curtis, *p* = 0.002; weighted UniFrac, *p* = 0.002) and AH + AR subjects (Bray Curtis, *p* = 0.002; weighted UniFrac, *p* = 0.001), as well as, separation between the AH + AR and the AH phenotypes (Bray Curtis, *p* = 0.035 5; weighted UniFrac, *p* = 0.038) or the AR group (weighted UniFrac*, p* = 0.038) (Figure 1D). No significant partition was evidenced between the AR and AH phenotypes. By comparing the different phenotypes to the distributions of the intra-group beta diversity, the results showed that the distance between individuals was significantly lower in diseased groups respect to the control one (Figure 1C). Same results were observed between the AH + AR and the AH and AR groups, while no difference were observed between groups with single pathology.

### A Stable and Quantitatively Dominant Core Microbiota Can Be Evidenced in the Anterior Nares of Children

**Figure 2:**
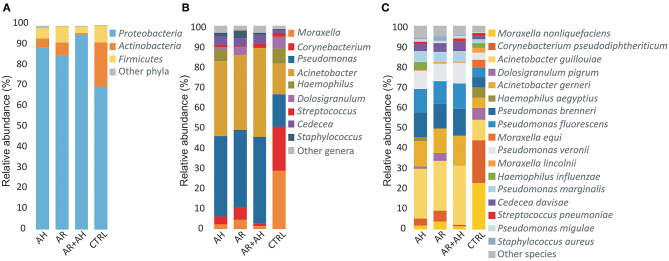
Color-coded barplots showing the average distribution of bacterial taxa at **(A)** phylum, **(B)** genus, and **(C)** species levels across different phenotypes. Only taxa for which a mean relative abundance = 1% was determined in at least one group, were reported in plots. Taxa were sorted respect to the descending order of the mean relative abundances in the CTRL group.

A total of 12 different phyla, 118 genera and 269 bacterial species were determined across all samples of which only 3 (25%) phyla, 9 (7.6%) genera, and 17 (6.3%) species presented a mean relative abundance ≥1% in at least one considered phenotype (Figure 2). *Proteobacteria* showed to be the most abundant phylum in all groups followed by *Actinobacteria* and *Firmicutes* with different extents in disease associated phenotypes and healthy individuals. Within *Proteobacteria*, the *Gammaproteobacteria* represented the class with the highest mean relative abundance in all groups (AH 89.2%, AR 84.9%, AH + AR 94.4%, CTRL 69.6%). At the genus level, a more variable distribution of taxa was demonstrated with healthy subjects showing *Corynebacterium* and *Moraxella* as the first two more abundant genera in the control group, while *Pseudomonas* and *Acinetobacter* revealed the higher mean relative abundance in diseased subjects. Furthermore, *Moraxella nonliquefaciens* and *Corynebacterium pseudodiphtericum* were the most abundant species in the control group while *Acinetobacter guillouiae* presented the highest mean relative abundance in all diseased groups followed by *Acinetobacter gerneri* and *Pseudomonas brenneri*.

The analysis of the prevalence of OTU in the overall studied population showed the presence of a core microbiota (OTU present in 100% of individuals). Such conserved component of microbiota, was composed by seven different OTUs that have been taxonomically assigned to bacterial species *A. guillouiae, A. gerneri, P. brenneri, Pseudomonas fluorescens, Pseudomonas veronii, Pseudomonas marginalis*, and *Cedecea divisae*. The core microbiota represented only the 1.6% of all identified OTUs while comprised the most part of sequences (71.1%; 2.351.589/3.307.515). When considering higher taxonomic levels, the core microbiota comprised bacteria all belonging to the phylum *Proteobacteria* and represented the 2, 6% (3/118) and 2, 6% (7/269) of identified genera and species, respectively.

### Differential Abundance Analysis of Taxa Evidences Core Microbial Groups Associated to Healthy State

**Figure 3:**
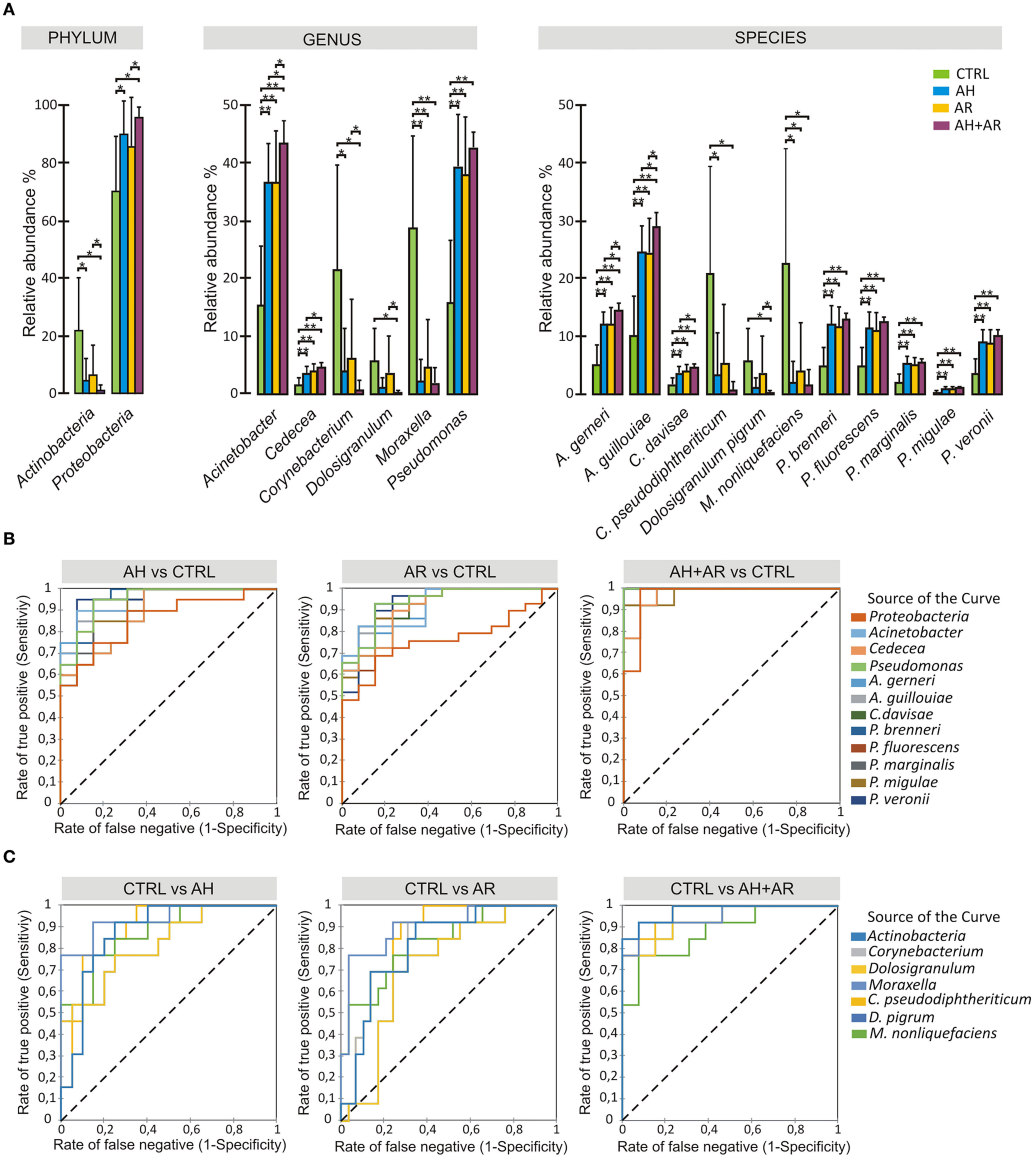
Differential abundance analysis and diagnostic power of most abundant taxa. **(A)** Color-coded barplots showing differential abundance analysis at phylum, genus and species levels performed by Kruskal-Wallis test followed by Games-Howell *post hoc* tests with Benjamini-Hochberg FDR correction to account for multiple comparisons. Only taxa showing significant differences among groups and for which a mean relative abundance ≥1% was determined in at least one group, are shown in plots. Values are expressed as mean ± SD. **(B)** ROC curve plots for taxa differentially abundant in diseased groups and in **(C)** control subjects. The areas under the ROC curves represent the specificity and sensitivity of the selected taxa able to discriminate AH, AR and AH+AR phenotypes from the control group. Dotted line represents the AUC = 0.5 (random classifier). *p ≤ 0.05; *p ≤ 0.001.

The analysis of the relative abundance among the different groups (Figure 3A) underlined the presence of differentially abundant taxa. At phylum level, *Proteobacteria* and *Actinobacteria* were significantly more abundant in diseased and control groups, respectively. Among *Proteobacteria, A. gerneri, A. gouillouiae, C. divisae, P. brenneri, P. fluorescens, P. marginalis, Pseudomonas migulae*, and *P. veronii* presented higher relative abundance in subjects affected by AH and/or AR respect to control group, while *C. pseudodiphtericum, Dolosigranulum pigrum*, and *M. nonliquefaciens* were significantly more abundant in control group. Interestingly, all core taxa at phylum, genus and species levels showed to be differentially abundant among phenotypes. The pairwise comparisons between diseased groups, revealed that differences in the relative abundance of taxa between AH and AR were never determined while, significant differences were found between the AH or AR phenotypes and the AH+AR one. Notably, for each considered taxa, the AR + AH group presented the most extreme values of relative abundances among groups. The findings obtained by the differential abundance analysis point out attention on highly conserved taxa that result to be enriched in to controls (Control Associated Taxa; CAT) or in diseased groups (Disease Associated Taxa; DAT). Furthermore, the differences found among diseased groups are in agreement with the results obtained by the β-diversity analysis respect to the driving of AR and AH toward a similar microbiota composition and the production of a sort of additive effect in presence of both pathologies. To evaluate if the differentially abundant taxa could discriminate between control and diseased subjects, the Receiver Operating Characteristic (ROC) curves, as well as the Area Under the Curve (AUCs), were calculated for CAT and DAT (Figure 3, Table S1). All considered taxa showed an AUC in a range of 0.73–1.0 with most of them presenting values ≥0.9. Such findings reveal a marked ability of CAT and DAT to classify individuals on the base of the presence/absence of the considered pathologies, suggesting the diagnostic potentials of analyzed taxa as well as the usefulness of nasal swabs as sampling tool.

### Microbial Taxa Associated to Nasal Health and Disease Are Differentially Correlated With Antigen-Specific Serum IgE and the Nasal Flow

**Figure 4:**
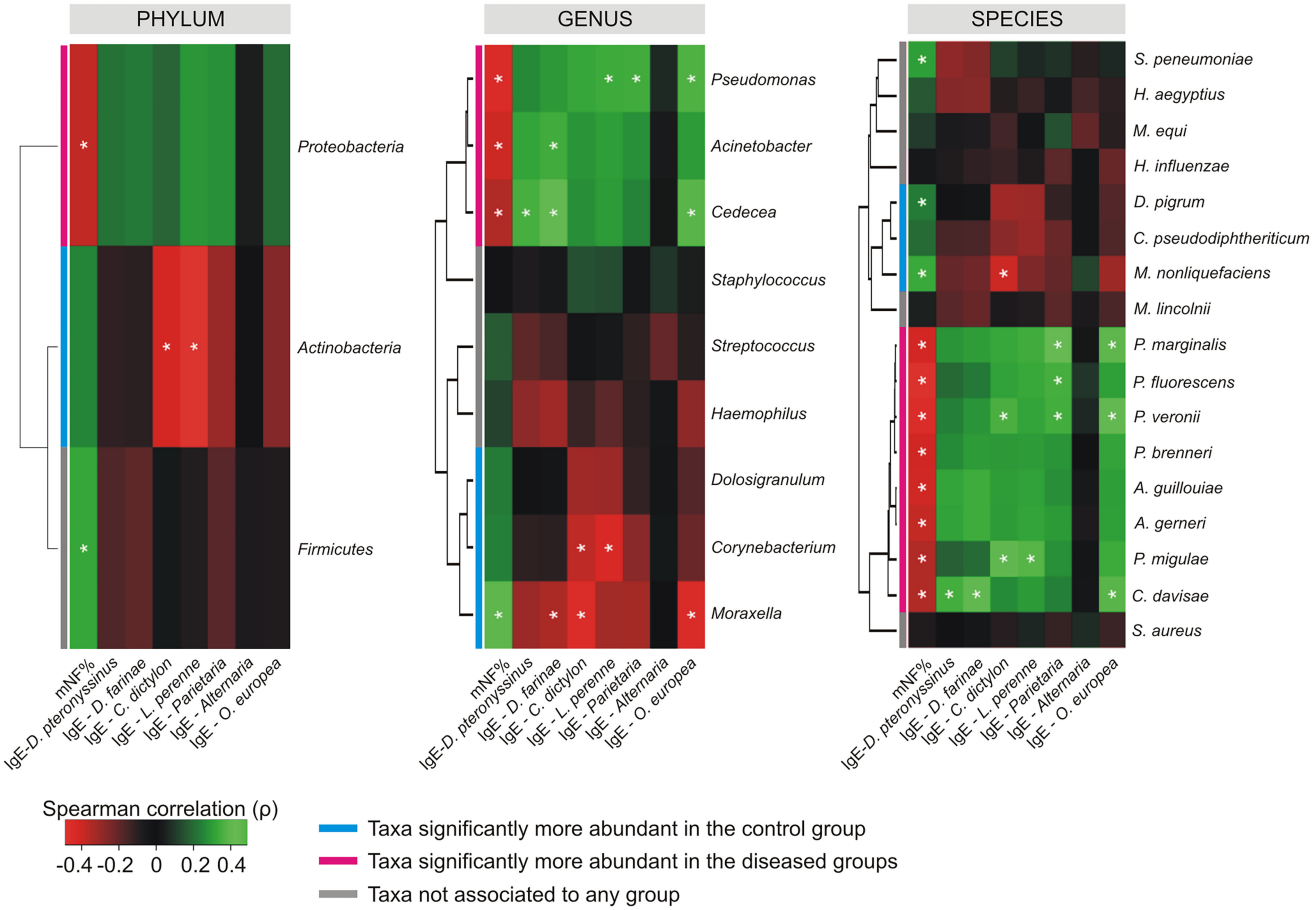
Cross-correlation heatmaps based on Spearman's correlation coefficients computed between the relative abundance of taxa ≥1% in at least one group and the values observed for mNF% and antigen-specific serum IgE across the whole population of studied subjects. The color scale represents values assumed by the Spearman's correlation coefficient (ρ) with green and red for positive and negative correlations, respectively. Taxa were ordered according to hierarchical simple-linkage clustering based on Spearman's coefficients computed on relative abundances (dendrogram on the left). The color coded bar indicates the differential association of taxa to groups according to results produced by the differential abundance analysis. A white asterisk indicates significant correlation at α level 0.05 after FDR correction for multiple comparisons.

The Spearman's rank correlation coefficient computed between the relative abundance of taxa, and the values collected for mNF%, and antigen-specific serum IgE across the whole population, revealed the presence of differential correlation patterns for CAT and DAT (Figure 4). Where present, only significant positive cross-correlations were observed between CAT and the nasal flow while only negative cross-correlations were determined between DAT and the same clinical variable. An opposite situation was observed for antigen-specific serum IgE levels relative to *D. pteronyssinus, D. farinae, C. dictylon, L. perenne*, and *O. europea* which presented only positive and negative correlation with DAT and CAT, respectively. No significant cross-correlations were determined between mNF% or antigen-specific serum IgE levels and the taxa not associated to any groups by the differential abundance analysis except for positive correlations between the nasal flow and both the phylum *Firmicutes* and the species *Streptococcus pneumoniae*.

### Network Analysis Reveals the Loss of Relationships Between Microbial Groups in Diseased State, as Well as, Peculiar Relationships Between Taxa Associated to Health and Disease

**Figure 5:**
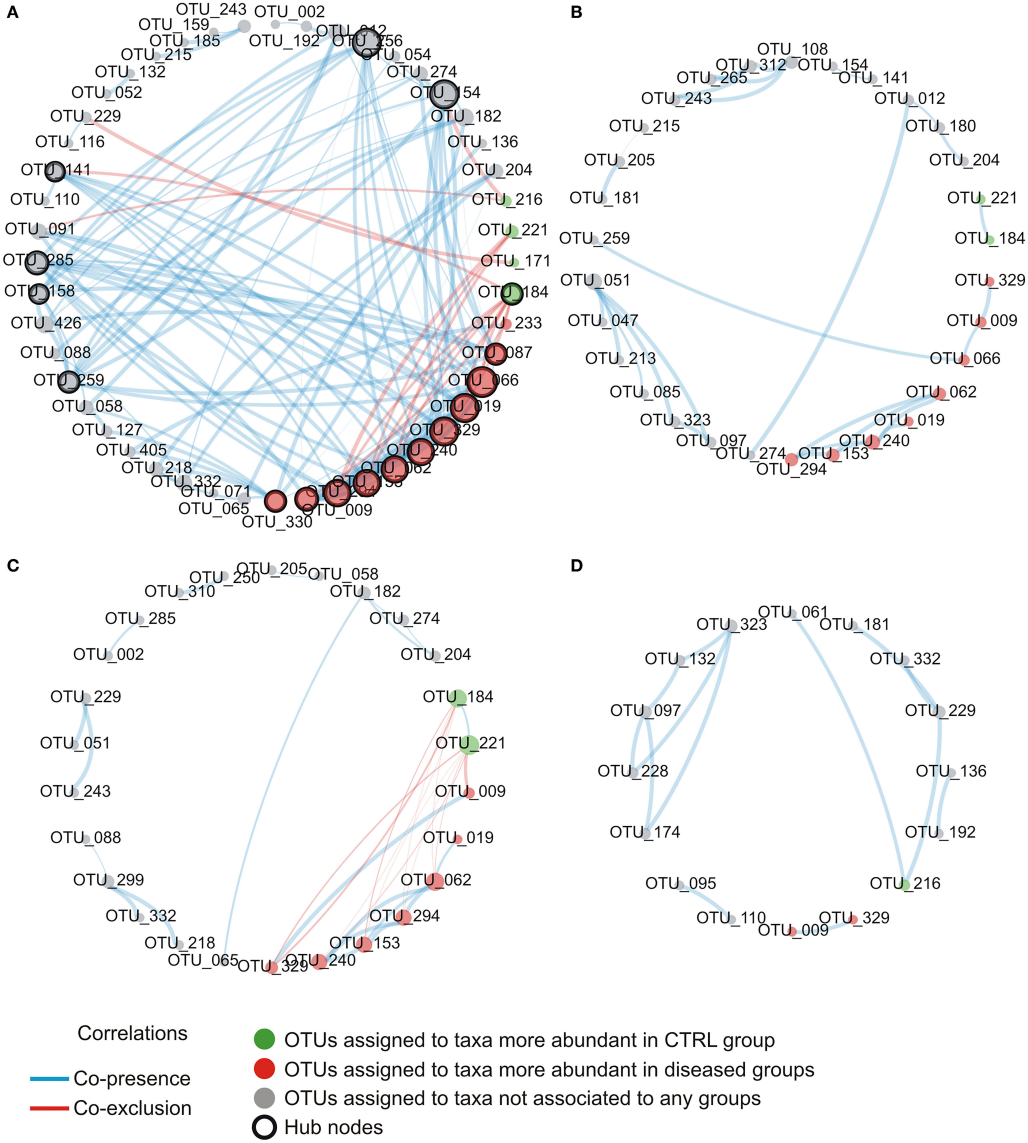
Intra-community network analysis taking into account OTUs presenting a mean relative abundance ≥0.01% across the whole population of samples. **(A)** CTRL, **(B)** AH, **(C)** AR, **(D)** AH+AR. The size of each node is proportional to the number of edges departing from it while the edge thickness is proportional to the strength of correlations.

explore microbial interactions within the nasal microbiota, a co-occurrence/co-exclusion analysis was carried out by using the CoNet software. In order to take into account only significant correlations, a combination of permutations and bootstrap were used. Microbial networks were evaluated for properties including centrality measures (Table 2). The network relative to the control group presented the best values respect to the number of participants (number of nodes) and connectivity (edge to node ratio, average number of neighbors, clustering coefficient, network density, number of connected components), while, a general reduction of nodes together with the loss of interactions, were found in diseased phenotypes with the most severe effect observed in the AH+AR group. The graphical representations of computed networks (Figure 5) show that only strong negative interactions were observed between the OTUs assigned to control-associated species and those assigned to species differentially enriched in diseased groups, while, where present, only positive correlations were evidenced within each group of taxa. Interestingly, OTUs assigned to CAT showed no positive correlations with each other, while analyzing the control group, such correlations appeared in AH and AR groups. Obtained results suggest that, within the nasal microbial community, synergistic relationships are present among potentially pathogenic taxa although with a different degree in each disease. In order to identify main taxa within microbial networks (Hubs), a degree based approach was used by considering, as important, nodes presenting a number of degrees equal or >50% of the highest number of connections presented by a single node in all networks.

**Table 2:** Topological properties of computed correlation networks.

**Network property**	**CTRL**	**AH**	**AR**	**AH + AR**
No. of nodes	48	30	26	16
No. of edges	133	29	32	13
Edge to node ratio	2.77	0.97	1.23	0.81
Network diameter	7	5	3	3
Connected components	4	7	7	5
Clustering coefficient	0.44	0.28	0.34	0.00
Average number of neighbors	5.54	1.93	2.46	1.62
Network density	0.19	0.07	0.09	0.11

Detailed information about the number of degrees and the determination as hubs for each node were provided as supplementary file (Table S2). In the control group, OTUs assigned to the disease-associated species *A. gerneri, A. guillouiae, Cedecea davisae, P. brenneri, P. fluorescens, P. marginalis, Pseudomonas migulae*, and *P. veronii* as well as to the health-associated taxa *C. pseudodophtericum*, resulted as main node of the microbial network. For all nodes within the correlation networks relative to AH, AR, and AH + AR groups, a number of degree smaller than the threshold was determined.

### Pathways Related to Human Pathogenesis Are Enriched in the Nasal Microbiota of Children Suffering of AH and AR

**Figure 6:**
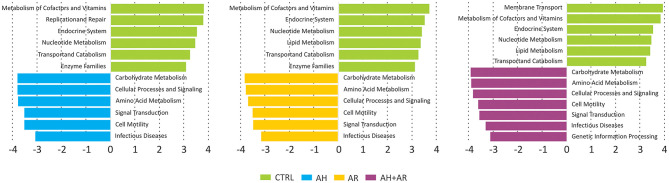
Results of LEfSe method performed on the relative abundances of KEGG pathways at L2 level obtained by reconstructing metagenomes with the PICRUSt algorithm. Pairwise comparisons were performed separately between each diseased groups and the control one. A FDR adjusted *p*-value ≤ 0.05, as well as an LDA score ≥3, were used as thresholds to identify significant features.

Functional metagenomic prediction, based on PICRUSt analysis and LEfSe results, showed significantly enriched KEGG pathways between diseased phenotypes and the control group at L2 level. As showed in Figure 6, all comparisons between diseased and control groups evidenced the presence of a similar pattern of differentially enriched gene functions comprising carbohydrate metabolism, amino acid metabolism, signal transduction. As expected, the diseased phenotypes resulted to be significantly enriched in pathways related to human pathogenesis.

## Discussion

Previous research has shown evidence for dysbiosis of the nasal microbiota in the context of AR (Choi et al., 2014; Lal et al., 2017; Wise et al., 2018), CRS (Ramakrishnan et al., 2015; Lal et al., 2017; Mahdavinia et al., 2018) and asthma (Durack et al., 2018; Fazlollahi et al., 2018), however still very little is known about AR and AH that are common conditions in children and are often associated with each other. Furthermore, available studies appear to be fragmented due to disease subgroups, phenotypes of included cases, age, sampling sites studied, as well as protocols used for sample collection and processing. The aim of this study was to explore, for the first time, whether and to what extent there might be differences in the nasal microbiota of healthy control subjects compared to patients with AR and AH. Although a limited sample size was analyzed, and the viral or fungal components of nasal ecosystem were not profiled, we found significant differences at various taxonomic levels among diseased groups and controls. Our data reveal that, respect to the healthy individuals, the presence of AH and/or AR is associated to alterations of the nasal microbiota composition with similar extent when only one of such disease is present. Overall results obtained for α and β-diversity showed that these diseases are associated to the trend to increase biodiversity as well as to limit both the intra-individual and intra-group variability. Furthermore, the results obtained by biodiversity analysis, suggest that the local inflammation common to AH and AR, through the alteration of both the environmental conditions and the available ecological niches, could limit the spectrum of microbial taxa able to colonize anterior nares. On the other hand, the dysbiotic environment could favor the acquisition of new specific bacterial groups, the overgrowth of selectively advantaged bacteria and the rise of variants among the same taxa. These hypothesized dynamics are not a novelty for the human microbiota. The tendency to the acquisition of peculiar taxa as well as to the overgrowing of potentially pathogenic bacteria has been also shown in the gut (Stecher et al., 2012; Brown et al., 2015) and oral associated microbiota (Lamont and Hajishengallis, 2015), in which, resident commensal microorganisms may become pathogenic when, the environmental changes, provided by the host tissue damage, create a microenvironment favorable to “inflammophilic” organisms. The relative abundance of phyla across the whole populations revealed to be in agreement with previously published papers in which *Proteobacteria, Actinobacteria*, and *Firmicutes* were reported as the most abundant bacterial groups in healthy children (Costello et al., 2009; Grice et al., 2009; Camarinha-Silva et al., 2012; Wos-Oxley et al., 2016; Birzele et al., 2017). However, the distribution of taxa showed clearly distinctive profiles between the control group and the diseased ones at different taxonomic levels. Contrariwise, among diseased phenotypes a more similar composition was observed with a slight deviance in presence of both pathologies. According to other studies (Camarinha-Silva et al., 2014; Prevaes et al., 2016) at the genus level, *Moraxella* and *Corynebacterium* represented the first two abundant genera in healthy subjects supporting the hypothesis about the main role of these two taxa in the nasal microbial community of healthy children. Conversely, the enrichment of the genera *Pseudomonas* and *Acinetobacter* in diseased subjects further supports their potential about their potential pathogenic role in human respiratory tract infections (Moradali et al., 2017; Wong et al., 2017). The determination of microbial groups shared by all individuals is usually considered pivotal in research fields concerning the human microbiota. In fact, the characterization of composition and dynamics of the core microbial components have deep implications on defining what could constitute a “normal” microbiota as well as, on the development of diagnostic models and therapeutic approaches applicable to diseases associated to microbiota alterations (Aguirre de Cárcer, 2018). The definition of a core microbiota constitutes a complicated affair in the context of microbiota studies mainly depending on the high variability of the human microbiota composition. Other hindering factors are linked to study-specific characteristics contributing to artificially increase the heterogeneity of data such as the inclusion of different sampling sites, the strength of the used analysis pipelines as well as the use of taxonomic levels. Particularly, taxonomic assignments are heavily biased even in well-sampled groups (Beiko, 2015) so the identification of the core microbiota on the base of taxonomy could not be adequate when considering downstream analyses and applications. Our results, clearly evidenced, at OTU level, the presence of a stable and quantitatively dominating core microbiota present in all studied subjects. However, surprisingly, the taxa belonging to the core microbiota are representative of bacterial species known to be also potential pathogens. Multiple studies found that anterior nares are an important habitat for clinically relevant pathobionts (Brugger et al., 2016), and emerging evidences show that airway microbiota may modulate local immune responses, thereby contributing to the susceptibility and severity of acute respiratory infection (Toivonen et al., 2019). Moreover, in urban areas, it appears that the increase in allergic respiratory disease is coupled by increasing atmospheric concentration of pollutants able to induce airway inflammation and airway hyper-responsiveness (Zou et al., 2018). Therefore, we could speculate that the core microbiota found in our study, in urban children could also been shaped by environmental factors, known to impact, resident bacterial communities. The differential analysis of the relative abundance of taxa revealed significant modifications of bacterial groups among phenotypes at different taxonomic levels. At genus and species level, the taxa significantly more present in the control group, were previously reported as dominant components of the natural microbiota of nares. In such environment, these taxa seem to promote more stable microbial communities and to prevent colonization by pathogenic bacteria (Biesbroek et al., 2014; Santee et al., 2016; Lappan et al., 2018). In particular, the genus *Dolosigranulum* has been associated to hinder the colonization of *Staphylococcus aureus* in the anterior nares (Liu et al., 2015) while *Corynebacterium* and *Moraxella* have been shown to be active against *S. pneumoniae* (Bomar et al., 2016) and associated to a lower frequency of upper respiratory tract infections (Santee et al., 2016). In the context of anterior nares, our results confirm the presence of specific bacterial groups in the health status, that were also associated to the AH and AR.

Our findings imply that these two pathologies could cause an increased instability of nasal microbial communities which could favor a shift toward pathogen-stable ecosystem. It is interesting to report that, despite the evidence about their beneficial properties, the genera *Corynebacterium* and *Moraxella* and the species *C. pseudodiphtericum* and *M. nonliquefaciens* have been previously associated with opportunistic infections within the upper respiratory tract, as well as, to an increased risk to develop diseases such as acute sinusitis (Biesbroek et al., 2014; Burkovski, 2015; Santee et al., 2016). This apparent discrepancy can be explained by the fact that within the human-associated microbiota, taxa could be composed by heterogeneous sub-populations comprising species/variants acting as pathobionts. Pathobionts, represent commensal bacteria able to exert a pathogenic behavior in response to altered environmental conditions like those presented by inflamed anterior nares (Gomes-Neto et al., 2017). Similar speculations may be extended to almost all DAT. In fact, although these taxa belonged to the stable microbiota shared even by control subjects, they comprised bacterial groups well-known for their ability to induce inflammatory responses. In the light of such results, these taxa should be considered as common components of the microbiota associated to the anterior nares rather than merely transient pathogens. However, in order to fully explore the complex behavior of such microbial groups, further studies, focused on the functional content of the nasal microbiota, are needed. Overall results provided by the analysis of the relative abundance of taxa underline, for the first time in children, that the compositional changes observed in AH and AR subjects, deeply involve the more intimate components of nasal microbiota. Furthermore, our study highlights that, depending on the health status of the subjects, single microorganisms could play multiple roles. This evidence has important implications in clinical aspects such as the development of therapeutic strategies. The analysis of ROC curves reveal that the relative abundance of CAT and DAT not only delineate a microbial signature of AH and AR but also, showed marked predictive potentials. These findings may provide a starting point for future research with the potential to improve patient outcomes. Furthermore, results obtained by our study candidate the nasal swab as easy, cheap, and efficient tool for sampling microbiota in the anterior nares. To elucidate microbial interactions within the nasal microbiota a co-occurrence/co-exclusion analysis was performed by considering each group separately. The topological properties determined for constructed networks strongly showed that, in the shift from a healthy state to the diseased condition, the microbial community undergoes a pauperization in terms of both, number of microbial taxa participating to the network and connectivity. Moreover, the analysis of the importance of nodes in computed networks showed a central role for different microbial taxa including almost all OTUs assigned to DATs. Such taxa were responsible for most part of the microbial interactions in the healthy status while their main role seems to be lost in diseased groups. These findings perfectly represent the “dysbiotic condition” in which the loss of the equilibrium within the microbial community is not only defined by quantitative modifications of taxa abundances but even by the disruption of the mutualistic interplay between microbes as well as, by the loss of the central role of key microbial components (Venturelli et al., 2018). Interestingly, peculiar correlation patterns have been evidence within and between DAT and CAT. These specific combinations of relationships could result from competition for the same trophic resources within the nasal ecosystem, or from mutualistic relations between metabolic-linked bacteria. In particular, synergistic correlations have been previously observed between *Corynebacterium* and *Dolosigranulum* (Laufer et al., 2011). It has been suggested that the production of lactic acid by *Dolosigranulum* makes the environment more favorable for *Corynebacterium* species (de Steenhuijsen Piters et al., 2015), which permit to explain the co-occurrences observed between OTU184 (*C. pseudodiphtericum*) and OTU221 (*D. pigrum*) in our study. Interestingly, no positive correlations were found among CAT in the healthy group while such relationships were observed in the AH and AR groups. Although we cannot exclude that the absence of positive relationships within CAT resulted from the use of too conservative statistical methods in calculating microbial networks, the positive correlations observed, only in diseased phenotypes, between these taxa, could evidence a tendency of the nasal microbiota to restore a healthy condition. The ability of the human-associated microbiota to maintains its equilibrium after an external perturbation, is known as the resilience phenomenon. Resilience describes the amount of stress that a system can tolerate before its homeostatic state shifts (Gunderson, 2000) and determines whether a particular perturbation will permanently shift its stable state or whether it will return to its initial homeostatic state. Such characteristic has been extensively demonstrating for the gut microbiota (Lozupone et al., 2012; de Steenhuijsen Piters et al., 2015; Greenhalgh et al., 2016; Sommer et al., 2017) while, although the stability of the nasal microbiota has been investigated by several studies (Meltzer et al., 2004; Frank et al., 2010; Ramakrishnan et al., 2013; Yan et al., 2013), its ability to respond to perturbations remain unclear. However, our data suggest a partition of the nasal microbiota in sub-populations of bacteria acting as equalizers in the microbial community. Balanced interactions between these groups of taxa could play a central role in keeping eubiosis as well as, to control the rise of potentially harmful subgroups of bacteria. Antigen-specific serum IgE are involved in allergic inflammation, especially in early-phase response, but it may also have a role in the late-phase allergic response (Galli et al., 2008). The positive and negative cross-correlations found between antigen-specific serum IgE levels and the relative abundance of different DAT and CAT respectively, showed that those microbial groups are differentially correlated to the level of sensitization to allergens. To date, only scarce data are available demonstrating a link between the microbiota composition and IgE levels. In a previous prospect study, Hyun and colleagues characterized the microbiota of the inferior turbinate of AR patients and healthy controls (Hyun et al., 2018). In the same study, the relationship of the mucosal microbiota with the presence of AR, as well as, the allergen-specific IgE levels was investigated. Although authors were not able to determine association between changes in the microbiota composition and the presence of AR or the levels of individual allergen-specific IgE levels, microbial dysbiosis together with the decrease of the phylum *Actinobacteria* was shown in presence of increased levels of total IgE. These results support our findings that the IgE-mediated inflammatory response characteristic for AR may influence microbial groups within the microbiota even though with potentially different dynamics along the upper airways. A prediction of functional profiles was performed by the PICRUSt algorithm which infer evolutionarily conserved functional gene capacity by starting from biomarker gene sequence data as 16S rDNA. Obtained results showed that the presence of AH and/or AR was associated to similar perturbations of functional pathways comprising signal transduction, amino acid metabolism, carbohydrate metabolism and gene functions related to infectious diseases (Figure 6). These findings are in line with those previously reported for inflammatory diseases of the upper respiratory tract (Pérez-Losada et al., 2015; Hyun et al., 2018) as well as in individuals with Crohn's disease and atopic dermatitis (Tong et al., 2014; Song et al., 2016) indicating that the alteration of microbial composition is coupled with the enrichment of specific gene functions related to pathogenicity. Notably, all analysis performed in our study evidenced that the co-presence of AH and AR seem to produce boosted effects on microbial composition, on relative abundance of the key microbial markers and on the interplay between microbial taxa. These stronger effects seem to overlap the coupling of reduced nasal flow and higher levels of antigen-specific serum IgE. It has been previously reported that inflamed tissues are often characterized by low levels of oxygen that, together with the altered interleukin secretion, induces hyperplasia of the goblet cells (Ding and Zheng, 2007; Jiao et al., 2016) and augmented secretion of mucus (Kim et al., 2014; Saieg et al., 2015; Jiao et al., 2016). These factors induce an increased thickness of mucus layer and lower levels of mucus flow with consequent mucus accumulation. Low availability of oxygenmay determine the remodeling of tissues through the upregulation of inflammatory pathways (Steinke et al., 2008; Nizet and Johnson, 2009), the reduced expression of antimicrobial proteins (Pahlman et al., 2015) and increased inflammation-associated tissue damages (Hoenderdos et al., 2016). Results obtained in our study suggest that the nasal flow, by influencing the availability of oxygen, could represent a secondary force supporting the local inflammation in shaping the microbiota composition and may constitute an additional risk factor for dysbiosis in the anterior nares. The positive and negative correlations of the mNF% with the relative abundance of CAT and DAT respectively, further evidence the role of nasal flow as an important driver able to shape the nasal microbiota by influencing its main microbial components. In conclusion, our results highlight the dysbiosis of the nasal microbiota as a characteristic condition of the nares ecosystem in pediatric patients suffering from the inflammatory diseases AH and AR. A highly conserved component of microbiota, as well as keystone taxa with promising diagnostic potential, have been identified. Interestingly, our study evidence that, in the nasal ecosystem, the inflammation-associated shifts of the microbiota seem to be characterized by changes in the ratio of the core components rather than the mere loss of microbial diversity. The identification of potentially pathogenic taxa as key bacterial groups, not only lead to review their contribute to the ecology of anterior nares of children, but also emphasize that the baseline composition of nasal microbiota could be conditioned by the environmental context such as living in urbanized areas.

## Data Availability Statement

All data generated or analyzed during this study are included in this published article (and its Supplementary Files). Raw data are available on NIH Sequence Read Archive (SRA) under the Bioproject ID PRJNA554533.

## Ethics Statement

The studies involving human participants were reviewed and approved by Bioethics Committee, Sapienza University of Rome. Written informed consent to participate in this study was provided by the participants' legal guardian/next of kin.

## Author Contributions

All authors listed have made a substantial, direct and intellectual contribution to the work and approved it for publication.

## Conflict of Interest

The authors declare that the research was conducted in the absence of any commercial or financial relationships that could be construed as a potential conflict of interest.

## Supplementary Material

Table S1 Detailed information about the ROC curves computed for CAT and DAT between control and diseased subjects. The Area Under Curve (AUC), its confidence interval at 95% (CI95%) and the statistical significance respect to the random classifier (*p*-value) were reported.

Table S2 Detailed information about the analysis of important nodes in computed microbial networks. For each node, the number of degrees, the percentage of maximum number of degrees among all networks and the node classification were reported. N/P taxa not present in the network.
